# Randomised Phase I/II trial assessing the safety and efficacy of radiolabelled anti-carcinoembryonic antigen I^131 ^KAb201 antibodies given intra-arterially or intravenously in patients with unresectable pancreatic adenocarcinoma

**DOI:** 10.1186/1471-2407-9-66

**Published:** 2009-02-25

**Authors:** Asma Sultana, Susannah Shore, Michael GT Raraty, Sobhan Vinjamuri, Jonathan E Evans, Catrin Tudur Smith, Steven Lane, Seema Chauhan, Lorraine Bosonnet, Conall Garvey, Robert Sutton, John P Neoptolemos, Paula Ghaneh

**Affiliations:** 1 Division of Surgery and Oncology, University of Liverpool, 5th Floor-UCD Building, Daulby Street, Liverpool L69 3GA, UK; 2 Department of Nuclear Medicine, Royal Liverpool University Hospital, Prescot Street, Liverpool L7 8XP, UK; 3 Department of Radiology, Royal Liverpool University Hospital, Prescot Street, Liverpool L7 8XP, UK; 4 Centre for Medical Statistics and Health Evaluation, University of Liverpool, Shelley's Cottage, Brownlow Street, Liverpool, L69 3GS, UK

## Abstract

**Background:**

Advanced pancreatic cancer has a poor prognosis, and the current standard of care (gemcitabine based chemotherapy) provides a small survival advantage. However the drawback is the accompanying systemic toxicity, which targeted treatments may overcome. This study aimed to evaluate the safety and tolerability of KAb201, an anti-carcinoembryonic antigen monoclonal antibody, labelled with I^131 ^in pancreatic cancer (ISRCTN 16857581).

**Methods:**

Patients with histological/cytological proven inoperable adenocarcinoma of the head of pancreas were randomised to receive KAb 201 via either the intra-arterial or intravenous delivery route. The dose limiting toxicities within each group were determined. Patients were assessed for safety and efficacy and followed up until death.

**Results:**

Between February 2003 and July 2005, 25 patients were enrolled. Nineteen patients were randomised, 9 to the intravenous and 10 to the intra-arterial arms. In the intra-arterial arm, dose limiting toxicity was seen in 2/6 (33%) patients at 50 mCi whereas in the intravenous arm, dose limiting toxicity was noted in 1/6 patients at 50 mCi, but did not occur at 75 mCi (0/3).

The overall response rate was 6% (1/18). Median overall survival was 5.2 months (95% confidence interval = 3.3 to 9 months), with no significant difference between the intravenous and intra-arterial arms (log rank test p = 0.79). One patient was still alive at the time of this analysis.

**Conclusion:**

Dose limiting toxicity for KAb201 with I^131 ^by the intra-arterial route was 50 mCi, while dose limiting toxicity was not reached in the intravenous arm.

## Background

Pancreatic cancer has an exceptionally poor prognosis with overall 5 year survival rates ranging from 3 to 5% [1-4]. The majority of patients present with advanced disease with a median life expectancy of 3 to 10 months [5]. Gemcitabine is the standard first-line agent for the treatment of advanced pancreatic cancer [6]. A recent randomised controlled trial has shown significant improvement in survival by the addition of capecitabine to gemcitabine compared to gemcitabine alone [7]. Other agents that add activity to gemcitabine are erlotinib [8] and the platins [7] but the advantage is small. In the light of the poor prognosis of this condition even with palliative chemotherapy, the search is on for better ways to treat this disease. Novel agents and newer routes such as regional delivery are being targeted, in the hope of finding a treatment with better efficacy and less toxicity than conventional chemotherapy.

One novel approach is to use monoclonal antibodies conjugated with radionuclides, resulting in better targeting of the tumour [9]. The radiation component has a bystander effect, with killing of adjacent unbound cells. The greater concentration of the drug within the tumour may have the advantage of lessening toxicity to normal tissue, the latter being a factor that limits the dosage and effectiveness of systemically administered agents [10].

Carcino-embryonic antigen (CEA), a glycoprotein, is a tumour-associated antigen and elevated levels are detected in the cell membrane of tumours derived from epithelium [11-14]. Monoclonal antibodies to this antigen have been employed in clinical trials for several applications, such as radio-immunotherapy, antibody-directed enzyme prodrug therapy and radio-immunoguided surgery [15-17].

Anti CEA monoclonal antibodies have been employed for radio-immunotherapy (RIT) in the treatment of colorectal cancer, both in the palliative and adjuvant settings [16,17]. One phase II trial of 30 patients, using anti CEA monoclonal antibody, bound to I^131^, concluded that this mode of treatment was safe and effective, with toxicity being limited to mild and transient leukopenia and thrombocytopenia [16]. Locoregional delivery of chemotherapy has been reported in both pancreatic cancer and colorectal liver metastases, with improved overall survival and reduced toxicity when compared to systemic chemotherapy [18,19] in randomised controlled trials.

CEA is overexpressed in over 90% of pancreatic cancers, and represents a potential target for immunotherapy [20], although no completed clinical trial has been reported in pancreatic malignancy so far [21]. We conducted this phase I/II trial employing targeted radioimmunotherapy for cancers of the head of the pancreas, using anti-CEA monoclonal antibody KAb201 radiolabelled with Iodine^131^, administered either intravenously or intra-arterially via the gastroduodenal artery. The rationale for inclusion of an intra-arterial arm was the presumed greater concentration of the study drug at the target site, with the possible translation into greater efficacy coupled with the advantage of reduced toxicity secondary to regional delivery.

## Methods

This study was open to recruitment from February 2003 to July 2005 at a single centre.

### Eligibility

Patients with locally advanced or metastatic adenocarcinoma of the head of the pancreas were eligible. The inclusion criteria were age > 18 years, histological or cytological proof, at least one confirmed and measurable tumour site in the head of pancreas, Karnofsky performance status (KPS) ≥ 70 and life expectancy of at least three months. Patients who had undergone prior treatment were enrolled into the trial, provided there was a month's gap between the radiotherapy/chemotherapy (preceding six weeks for nitrosoureas).

Patients were excluded if there was haematological impairment, worsening hepatic impairment or significant renal dysfunction. Other exclusion criteria were known immunological reactions to previously administered antibodies, proteins or iodine, previous external beam radiotherapy to maximal tolerable levels to any critical organ and treatment with any other clinical trial medication within the preceding three months.

Following confirmation of eligibility, patients were randomised to receive the study drug by either the intra-arterial or intravenous route, using computer generated random numbers. The study was not blinded.

### Monoclonal antibody (MAb)

KAb201 is a human-sheep chimaeric monoclonal antibody [22] against CEA, produced by a pharmaceutical company (and study sponsor) Xenova Biomedix [23]. The linking of the radioisotope to the MAb helps detect and treat potential sites of disease. Iodine^131 ^was chosen as its half-life is close to the expected retention of the MAb at the tumour site, its β emission can kill tumours over a range of up to 40 cell diameters and its γ emission can help in imaging the biodistribution.

An earlier phase I study carried out on 10 patients with metastatic colorectal cancer using KAb201 with I^131 ^found the study drug to be well tolerated, with no drug related adverse events, good localisation at the tumour site and no antigen response in 9 patients [24].

Following this pilot study, the current phase I/II trial was designed for pancreatic cancer after an in vitro study, which yielded a sensitivity of 83% and specificity of 95% for staining with Kab 201. In vitro studies had been conducted by Xenova Biomedix and these showed specificity of the antibody for tumour tissue but not for normal tissue.

Radiolabelling for the pretherapy dose was done at the local Nuclear Medicine department, using the Iodogen method. Each 1 mg of KAb201 was labelled with 10 mCi of I^131^, prepared up to 24 hours before administration. Quality control was assessed using Mini TLC, aiming for labelling efficiency of > 60%. The therapeutic dose was prepared by Vrije University, Amsterdam, Netherland. The activity of the I^131 ^KAb 201 was assessed by the local Nuclear Medicine Department prior to administration. The stability of the radio labelling was > 90%.

### Study design

Following informed consent, patients underwent clinical evaluation, blood tests for haematological and biochemical assessment, tumour markers (CEA and CA19-9 levels), baseline evaluation of anti-sheep (HASA) and anti-chimaeric (HACA) antibody and radioactivity levels. Tumour size and extent were assessed by multi-detector computed tomography (CT) of the chest, abdomen and pelvis and/or F^18 ^positron emission tomography (PET).

Pre therapy dose with 2 mCi of I^131^-Kab201 was administered via the intended therapeutic route [25]. Intra-arterial drug delivery was either through a temporary catheter inserted at angiography using a 2.5 French prograde microcatheter (Terumo, Belgium) into the gastroduodenal artery or via a permanent catheter, a Port-A-Cath single-lumen implantable vascular access system (SIMS Deltec, Inc., St. Paul, Minnesota, USA), which was inserted into the gastroduodenal artery in patients who required palliative bypass surgery. Intravenous drug delivery was via a standard intravenous line (20 or 22 gauge Venflon). Twenty-four hours later, gamma camera static and single photon emission computerised tomography (SPECT) scans were performed to check for uptake of I^131 ^in the tumour. This pre therapy check scan was done to assess for uptake of I^131 ^KAb201 in the primary and/or secondary tumour. If the scan was positive, the patient received the therapy dose 5–7 days later and if there was no uptake, the patient was withdrawn from the trial.

At each dose level, six patients were to be treated, three patients per administration route (intravenous or intra-arterial). Following the therapy dose, patients were isolated for at least five days, in keeping with local radiation safety measures. Repeat gamma camera and SPECT scans were performed a week after treatment to assess localisation of I^131 ^KAb201 post-therapy.

Patients were followed up 2, 5, 7, 11, 14, 28, 42, 60 and 90 days post- treatment. The blood tests undertaken during follow up are summarised in Table 1. Analyses of blood results and urinalysis were done at a single independent laboratory (Pivotal Laboratory, York) except for radioactivity levels, which were evaluated in the local Nuclear Medicine Department of the Royal Liverpool University Hospital.

**Table 1:** Blood test schedule following treatment with I^131 ^KAb201

Blood test	Time points
Haematology	Day of treatment and days 5, 7, 11, 14, 28, 42, 60 and 90 post treatment
Biochemistry	Day of treatment and days 5, 7, 11, 14, 28, 42, 60 and 90 post treatment
Pharmacokinetics and radioactivity levels	Days 1, 2, 5, 7, 11, 14, 28, 42, 60 and 90 post treatment
HASA/HACA	Days 14, 28, 42, 60 and 90 post treatment
Serum CEA/CA19-9	Days 28, 60 and 90 post treatment
CEA complexing assay	6 hours post dosimetry

HASA = Human antisheep antibody; HACA = Human anti-chimaeric antibody; CEA = Carcino-Embryonic antigen

CT and PET scans were repeated at 1 and 3 months post treatment. Response was evaluated using the Response Evaluation Criteria in Solid Tumours (RECIST) criteria [2,26]. If progressive disease was seen on the one month post treatment scans, patients were allowed to withdraw from the trial treatment and be referred for palliative chemotherapy. These patients were followed up for ongoing toxicity, if any, as well as survival.

### Primary and secondary endpoints

The primary endpoints were safety, tolerability and level at which dose limiting toxicity occurred. The secondary endpoints were to assess the pharmacokinetics, antigenicity, efficacy and overall survival.

Safety was tolerability were assessed by clinical evaluation, Karnofsky performance status, urinalysis and blood tests (Table 1) and adverse events (AE) were graded by the Common Toxicity Criteria (CTC) version 2 [27].

Dose limiting toxicity (DLT) was defined as grade 4 neutropenia lasting for 7 or more days, febrile neutropenia, platelet count < 25 × 10^9^/L or counts between 25 × 10^9^/L to 50 × 10^9^/L associated with bleeding requiring transfusion, grade 3 or greater nausea despite adequate anti-emetics and any other grade 3 or 4 adverse events. If a DLT occurred in one of three patients, then a further three patients were treated at that dose level and route. Dose escalation was stopped if DLTs were seen in two or more patients.

Pharmacokinetics of both the monoclonal antibody KAb201 (frozen serum samples sent for analyses to Huntingdon Life Sciences, Cambridgeshire, UK) and radioiodine were studied (count done at local Nuclear Medicine department, using a gamma camera calibrated for I^131^), while antigenicity was assessed by estimating the Human anti-sheep antibody (HASA) and Human anti-chimaeric antibody (HACA) levels (frozen serum samples sent for analyses to Huntingdon Life Sciences, Cambridgeshire, UK).

### Statistical Analyses

The number (and percentage) of patients experiencing each adverse event were tabulated along with the number (and percentage) of occurrences of each event. Time to occurrence of first haematological toxicity was calculated from date of pre therapy dose to date of haematological toxicity (all grades) or the date of the last follow-up if censored. Overall survival was calculated from the date of randomisation to death or the censor date. Time to event data were analysed using the method of Kaplan and Meier [28] and presented graphically with median (95% confidence interval) data. The log rank test was used to assess differences across groups according to the route of administration, KPS, tumour stage (locally advanced versus metastatic disease) and prior treatment. A post-hoc analysis exploring the effect of baseline body surface area (BSA in cm^2^) on time to haematological toxicity (all grades) was undertaken using a Cox proportional hazards regression model.

Pharmacokinetics of KAb201 and I^131 ^were assessed using a single compartment model with instantaneous intravenous bolus and first order elimination rate to model both the therapeutic dose and radioactivity plasma concentrations. Area under the curve, Cmax, volume of distribution and rate of elimination were estimated.

Overall response rate (any partial or complete response) was summarised as a number/percentage.

### Conduct of the study

Written informed consent was obtained from all patients prior to entry to the study. The study was conducted according to International Conference on Harmonisation (ICH) on the topic Good Clinical Practice (GCP) guidelines. Ethical approval to carry out this study was given by the Liverpool Local Research Ethics Committee. The trial was conducted to conform to the principles of the Declaration of Helsinki.

## Results

### Patient characteristics

Twenty five patients were screened between February 2003 and July 2005. Six patients were screening failures and the other 19 patients were randomised, nine to the intravenous arm and 10 to the intra-arterial arm (Table 2). There was no loss to follow up.

**Table 2:** Patient characteristics (randomised patients)

	Intra-venous (n = 9)	Intra-arterial (n = 10)	Total (n = 19)
**Age, years**			
median ((25th, 75th centile) min, max	60 (59,60) 53–66	59 (55,63) 47–67	60 (57,63) 47–67
**Karnofsky Performance Status**			
Score ≤ 80	3 (33%)	7 (70%)	10 (53%)
Score ≥ 90	6 (67%)	3 (30%)	9 (47%)
**Stage**			
IVa	2 (22%)	6 (60%)	8 (42%)
IVb	7 (78%)	4 (40%)	11 (58%)
**Previous treatment**			
	3 (33%)^φ^	1 (10%)^ξ^	4 (21%)
**Time from diagnosis to randomisation, days**			
median ((25th, 75th centile)	84 (60,203)	37 (16,115)	77 (25,177)
min, max	27–316	2–256	2–316

^φ ^one patient each had received: gemcitabine plus oxaliplatin 6 cycles, gemcitabine 8 cycles and gemcitabine 7 cycles

^ξ^gemcitabine 3 cycles.

Four patients had received prior chemotherapy, three of whom had progressed on this prior treatment. One patient had regression of disease following chemotherapy (degree of response not evaluated) prior to enrolment onto the trial. Median time from diagnosis to entry into trial was 77 days (range = 25 to 177 days), and in those with previous treatment, this was 247 days (range = 199 to 311 days).

### Treatment summary

Seventeen patients received the pre therapy dose, nine by the intravenous route and eight by intra-arterial route (three via Port-A-Cath and five via a temporary catheter placed at angiography). Two patients could not undergo pre therapy dose due to problems with I^131 ^availability. Pre therapy scan uptake was seen in 16 out of 17 patients with lack of uptake occurring in one patient in the intra-arterial group. This patient was hence not given the therapy dose and was excluded from toxicity and efficacy analysis. Post-therapy uptake was seen in all 18 patients.

### Primary end point

**Figure 1:**
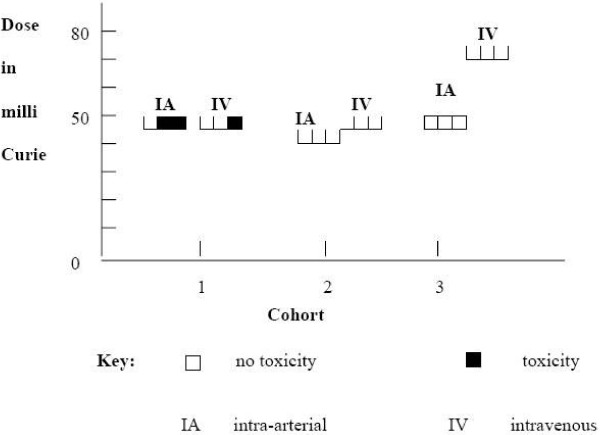
**Dose limiting toxicity**. In the intra-arterial arm dose limiting toxicity (DLT) was observed in two of three patients at 50 mCi in the first cohort and in none of the three patients each in both the second cohort (45 mCi) and the third cohort (50 mCi). In the intravenous arm, one out of three patients experienced DLT at 50 mCi and none out of the three patients each in the second (50 mCi) and third cohorts (75 mCi) suffered a DLT. In summary, 2/6 patients experienced DLT in the intra-arterial arm (MTD reached at 50 mCi) and 1/6 patients in the intravenous arm (MTD not reached).

Dose limiting toxicity (DLT) was observed in none out of three patients at 45 mCi and two out of six patients at 50 mCi in the intra-arterial arm and one out of six patients at 50 mCi and none out of three patients at 75 mCi in the intravenous arm (Figure 1).

The most common adverse event was haematological toxicity (all grades), seen in 17/18 patients (8/9 in the intra-arterial arm and 9/9 in the intravenous arm). One patient received treatment with granulocyte monocyte colony stimulating factor for febrile neutropenia. The median (95% Confidence Interval) time to occurrence of haematological toxicity was 24 (12 to 40) days, with an earlier onset in the intra-arterial arm (21 (12 to 34) days) compared to the intravenous arm (35 (12 to 42) days), although this difference was not statistically significant (log rank p = 0.92). A Cox regression model suggested a significant (p = 0.022) relationship between body surface area in cm^2 ^and time to first haematological toxicity (all grades), with a hazard ratio (HR) of 0.97 (95% CI = 0.949, 0.997) for a unit increase in cm^2 ^of BSA i.e. a higher BSA had a reduced hazard of haematological toxicity at any time. Some of the other grade 3 or 4 adverse events are detailed in Table 3.

**Table 3:** Grade 3 or 4 drug related adverse events

Event	Intra-arterial (n = 9)	Intra-venous (n = 9)	Total (n = 18)
Lymphopenia	3	2	5
Thrombocytopenia	4	2	6
Leukopenia	4	0	4
Neutropenia	3	0	3
Sepsis	1	1	2
Vomiting	1	1	2
Alanine aminotransferase	1	0	1
Anaemia	0	1	1
Anorexia	0	1	1
Aspartate aminotransferase	1	0	1
Blood alkaline phosphatase	1	0	1
Febrile neutropenia	1	0	1
Haematemesis	0	1	1
Neutrophilia	1	0	1
Thrombosis	1	0	1

### Secondary Endpoints

#### Pharmacokinetics and Radioactivity

**Figure 2:**
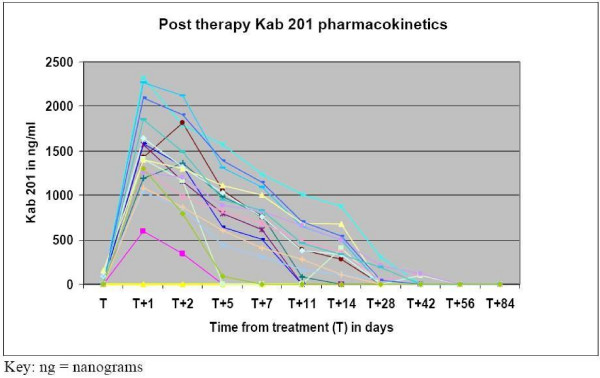
**Pharmacokinetics- Post treatment with KAb201**. The pharmacokinetics of KAb201 per individual patient (n = 18). After administration of the therapy dose, maximal levels (ng/ml) in blood were seen up to 5 days post treatment, with subsequent gradual decline and undetectable level by 1–2 months.

**Figure 3:**
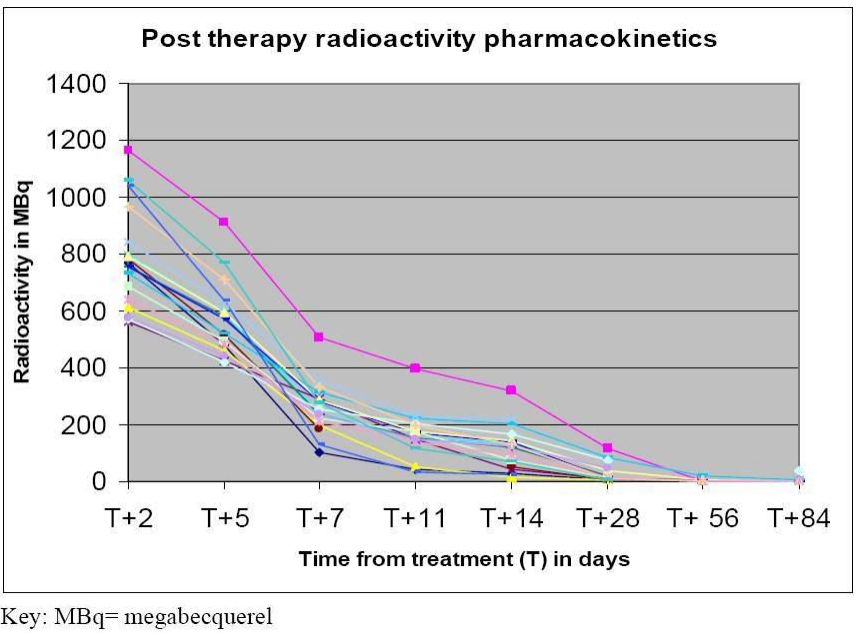
**Pharmacokinetics- Post-therapy dose blood radioactivity of I^131 ^levels**. The pharmacokinetics of I^131 ^per individual patient (n = 18). Radioactivity levels (MBq = mega bequerel) following administration of the therapy dose were high in the first five days, with levels gradually declining thereafter, to near undetectable levels in blood by 2 months.

Following administration of the pre therapy dose, KAb201 levels peaked at 10 minutes post administration, then declined steadily and levels were undetectable by one week. After the therapy dose, maximal activity was seen up to 5 days post treatment, with subsequent gradual decline and was undetectable by 1 to 2 months (Figure 2). Radioactivity levels changes were similar to the study drug pharmacokinetic data (Figure 3).

Employing the single compartment model, the population volume of distribution (V) was similar in the intra-arterial and the intravenous arms (Table 4 and Table 5) for both the therapy dose of KAb201 and radioactivity. The population elimination rate was significantly higher (p < 0.001) for the intravenous group compared with the intra-arterial group for both the therapy dose of KAb201 and radioactivity level.

**Table 4:** Pharmacokinetic analysis- Population estimates for the therapeutic dose of I^131 ^KAb201.

Parameter		Intra-arterial group	Intravenous group
Volume of distribution	mean (SE)	0.0032 (0.0004) L	0.0031 (0.0003) L
Elimination rate	mean (SE)	0.0944 (0.0095) litres/hour	0.1498 (0.0176) litres/hour
Area under Curve	mean (range)	15801.0 (5673.4, 30011.2) ng/ml	12411.3 (4748.3, 20713.8) ng/ml
Cmax	mean (range)	1488.7 (644.5, 2328.5) ng/ml	1812.7 (1002.8, 2600.3) ng/ml

SE = standard error; L = litre; h = hour; ng = nanogram; ml= millilitres

**Table 5:** Pharmacokinetic analysis- Population estimates for radioactivity following administration of the therapeutic dose of I^131 ^KAb201.

Parameter		Intra-arterial group	Intravenous group
Volume of distribution	mean(SE)	0.0600 (0.0048) L	0.0611 (0.0024) L
Elimination rate	mean(SE)	0.1461 (0.0179) litres/hour^1^	0.1992 (0.0224) litres/hour
Area under Curve	mean (range)	5509.0 (3245.3, 11049.7) ng/ml	4854.9 (3482.6, 6272.5) ng/ml
Cmax	mean (range)	806.0 (608.8, 1191.4) ng/ml	982.2 (754.4, 1386.9) ng/ml

SE = standard error; L = litre; h = hour; ng = nanogram; ml= millilitres.

#### Antigenicity

HACA antibodies developed in all 16 assessable patients. In two patients who could not be assessed, the sample was insufficient for analysis in one and in the other only a screening sample was available.

17/17 (100%) patients developed HASA antibodies. One patient could not be assessed for this parameter as only the screening sample was available.

#### Efficacy

Eighteen patients were assessable for efficacy analyses. There was one partial response, one stable disease, and 16 patients progressed. This yields an overall response rate of 6% and disease control rate of 11% (Disease control rate = partial response+ stable disease).

#### Overall Survival

For all 19 patients, median (95% CI) overall survival was 5.2 (3.3 to 9) months. There was no significant overall survival difference between the intravenous and intra-arterial arms (log rank p = 0.79). One patient was still alive at the time of this analysis (08 May 2006), at 13 months post-therapy.

**Figure 4:**
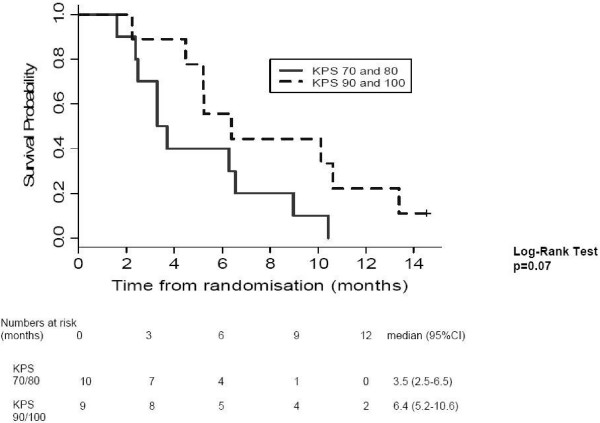
**Overall survival stratified by Karnofsky Performance Status**. This is a Kaplan Meir survival curve of all 19 patients, stratified by their Karnofsky Performance Status i.e. KPS 90/100 versus KPS 70/80. The trend towards improved survival in the higher KPS group was not statistically significant (log rank p = 0.07).

Patients with KPS scores of 90/100 survived longer than those with KPS of 70/80 (log rank p = 0.07; Figure 4). There was no difference in survival between patients with locally advanced and metastatic disease (log rank p = 0.93) and in those with or without prior treatment (log rank p = 0.34).

## Discussion

The maximum tolerated dose of 50 mCi by the intra-arterial arm in the present study is difficult to compare with the results of other studies, as we did not dose patients based on their body surface area. Being a phase I exploratory study, we used pre-specified dose levels in terms of mCi. However, on post-hoc review of the dose based on body surface area, patients in whom dose limiting toxicity was seen i.e. those administered 50 mCi by the intra-arterial route received a median dose of 27.5 mCi/m^2^. This is lower than the maximum tolerated dose of 60 mCi/m^2 ^reported by Behr et al [16], as well as the 40 mCi/m^2 ^found by Hajjar et al [29].

Surprisingly, despite the loco- regional nature of delivery of KAb201, systemic toxicity occurred. Directly labelled monoclonal antibodies are known to have a relatively low level of uptake in solid tumours, and additionally hampered by heterogeneous deposition of the antibody as well as radiation doses in tumour tissue [30]. The unbound radiolabelled KAb 201 circulating systemically is likely to be responsible for the toxicity observed.

By the intravenous route, a larger dose of radiation, up to 75 mCi at close of trial, equating to a median of 45 mCi/m^2^, could be delivered without causing dose limiting toxicity. The haematological toxicity seen is in keeping with previous reports, which also reported myelotoxicity to be the main dose limiting factor [16,31,32]. Dosimetry based planning of treatment and pretargeting may minimise this problem [31]. Since the aim of this phase I trial was to determine the MTD for KAb 201 with I^131 ^and the therapy doses were planned as per protocol, we did not use formal dosimetry.

A poor correlation between the toxicity grade and administered radioactive dose has been reported, leading to the conclusion that factors other than the total administered activity or the bone marrow dose are important [32]. The incidence of systemic toxicity in the intra-arterial arm implies that despite the loco regional delivery, there is spill over into the systemic circulation, supported by the finding of similar volume of distribution in both arms. The earlier occurrence of haematological toxicity in the intra-arterial arm could be linked to the slower rate of elimination seen in this arm, compared to the intravenous arm.

The similar pharmacokinetics of KAb201 and I^131 ^implies it was appropriate to combine these two agents, as their decline runs in parallel. In view of the fact that the levels reach near undetectable levels by 6–8 weeks in most patients, this time point, rather than the 3 months chosen at start of study, may be more appropriate for either a repeat dose or commencement of palliative treatment off-trial.

The antigenic response seen in the majority of patients to both the sheep and chimaeric component of the antibody limits the possibility of repeat dosing, as this may either lead to hypersensitivity reactions or complexing with circulating antibody, making it difficult or impossible to maintain effective therapeutic levels [15]. Repeat dosing using high affinity humanised monoclonal anti-CEA antibody have been reported [16,33].

Similar to our antigenicity result, Ritter et al, despite using a humanised murine monoclonal antibody, detected human anti-human antibodies (HAHA) in 63% of patients treated with repeat dosing [33]. They suggest that monitoring the type of antibody helped them single out patients at risk for transfusion-related adverse events, as those who developed type I HAHA (characterised by early onset, with levels peaking after 2 weeks and declining thereafter) did not develop infusion-related adverse events, unlike patients with type II antibodies (onset delayed, with levels increasing following repeat dosing).

The overall response rate of 6% is similar to that reported using single agent gemcitabine by Burris et al (5.4%) [6], as well as in two other large studies by Cunningham et al (7%) [34] and Moore et al (9%) [8].

The effectiveness of monoclonal antibodies has been limited by low quantitative delivery to tumours, poor diffusion from vasculature into tumour and biodistribution to normal organs [10]. Although tumour vessels have attributes that favour movement of molecules across the vessels such as wide inter-endothelial junctions, large number of fenestrae and discontinuous or absent basement membrane, these are offset by the high interstitial pressure and low microvascular pressure that may retard extravasation of molecules, particularly into large tumours [35]. Other factors limiting efficacy may be inherent radio-resistance and heterogenous expression of CEA [36].

Our study's median overall survival of 5.2 months is comparable to that achieved by single agent gemcitabine in several trials of 4.0–5.2 months [6,37,38], and somewhat inferior to the median survival of 6.0 to 7.3 months in the single agent gemcitabine arm in some others [34,39-41]). Survival with I^131 ^KAb201 may be boosted by combination with chemotherapy, which may also help radiosensitize the tumour. The chemotherapy suggested, based on a recent meta-analysis, is gemcitabine based combination chemotherapy [7]. Alternatively, to avoid further increasing marrow toxicity, combination with erlotinib may help improve its effectiveness.

## Conclusion

In summary, KAb201 with I^131 ^demonstrated tumour targeting, with haematological toxicity of varying degrees. The intra-arterial route did not confer any additional survival benefit or reduction in toxicity over the intravenous route, with a dose limiting toxicity at 50 mCi. Survival and efficacy data is comparable to the median survival and efficacy seen with single agent gemcitabine. The antigenic response observed with KAb 201 may raise questions over its suitability for repeated dosing. Investigation of the type of antibody response (type I or type II) in future studies may lead to the ability to predict those patients who are likely to suffer a transfusion related adverse event on repeat dosing. Humanisation of the antibody instead of the current human sheep chimaeric construct may reduce the immunogenicity.

Although RIT has been successful in the setting of lymphoma, the low therapeutic index observed with the one stage RIT approach have failed to produce equivalent antitumour effects in the more radio resistant solid tumours (referenced in [42,43]. As the tumour uptake, and thereby the radiation dose, is inversely related to tumour size [31], KAb201 may be a viable option in small volume metastatic disease. In view of the low toxicity seen in the intravenous arm, allowing dose escalation of up to 75 mCi, it may be feasible to combine KAb201 with I^131 ^given by the intravenous route with chemotherapy, as has been demonstrated in a preclinical study [44], or erlotinib, to improve efficacy.

## Abbreviations

AE: adverse event; BSA: body surface area; CEA: carcino-embryonic antigen; CI: confidence interval; CT: computed tomography; CTC: Common Toxicity Criteria; DLT: dose limiting toxicity; HACA: human anti-chimaeric antibody; HAHA: human anti-human antibody; HASA: human anti-sheep antibody; I^131^: iodine 131; HR: hazard ratio; KPS: Karnofsky performance status; MBq: mega Bequerel; mCi: milli Curie; MTD: maximum tolerated dose; PET: positron emission tomography; RECIST: Response Evaluation Criteria in Solid Tumours; RIT: radioimmunotherapy; SPECT: single photon emission computerised tomography scans.

## Competing interests

This study was sponsored by a pharmaceutical company Xenova Biomedix, now Avaant Pharmaceuticals.

## Authors' contributions

AS and SS enrolled patients in the clinical protocol. SV administered the radionuclide and interpreted the pre-treatment and post therapy gamma camera and SPECT scans. JEE performed the angiographic placement of microcatheter for patients receiving the study drug intra-arterially, and with CG, interpreted the CT/MRI scans. CTS and SL performed the statistical analyses. JPN, PG, MR, SC, LB and RS facilitated recruitment and management of patients. JPN was the primary investigator and PG was co-investigator. AS, SV, PG and JPN were involved in drafting the manuscript. All authors have read and approved the final manuscript.

## Pre-publication history

The pre-publication history for this paper can be accessed here:

http://www.biomedcentral.com/1471-2407/9/66/prepub
